# Towards the Improvement of Soil Salinity Mapping in a Data-Scarce Context Using Sentinel-2 Images in Machine-Learning Models

**DOI:** 10.3390/s23239328

**Published:** 2023-11-22

**Authors:** J. W. Sirpa-Poma, F. Satgé, E. Resongles, R. Pillco-Zolá, J. Molina-Carpio, M. G. Flores Colque, M. Ormachea, P. Pacheco Mollinedo, M.-P. Bonnet

**Affiliations:** 1 ESPACE-DEV, Univ Montpellier, IRD, Univ Antilles, Univ Guyane, Univ Réunion, 34093 Montpellier, France; paula.pacheco-mollinedo@ird.fr (P.P.M.);; 2 Universidad Mayor de San Andrés, Instituto de Hidráulica e Hidrología, La Paz, Bolivia; rpillco@umsa.edu.bo (R.P.-Z.); jamolinacarpio@gmail.com (J.M.-C.); 3 HSM, Univ Montpellier, CNRS, IRD, 34093 Montpellier, France; eleonore.resongles@ird.fr; 4 Universidad Mayor de San Andrés, Instituto de Investigaciones Químicas, La Paz, Boliviamormachea@fcpn.edu.bo (M.O.)

**Keywords:** Sentinel-2, machine-learning, soil salinity, data scarcity, Random-Forest, Support Vector Machine

## Abstract

Several recent studies have evidenced the relevance of machine-learning for soil salinity mapping using Sentinel-2 reflectance as input data and field soil salinity measurement (i.e., Electrical Conductivity-EC) as the target. As soil EC monitoring is costly and time consuming, most learning databases used for training/validation rely on a limited number of soil samples, which can affect the model consistency. Based on the low soil salinity variation at the Sentinel-2 pixel resolution, this study proposes to increase the learning database’s number of observations by assigning the EC value obtained on the sampled pixel to the eight neighboring pixels. The method allowed extending the original learning database made up of 97 field EC measurements (OD) to an enhanced learning database made up of 691 observations (ED). Two classification machine-learning models (i.e., Random Forest-RF and Support Vector Machine-SVM) were trained with both OD and ED to assess the efficiency of the proposed method by comparing the models’ outcomes with EC observations not used in the models´ training. The use of ED led to a significant increase in both models’ consistency with the overall accuracy of the RF (SVM) model increasing from 0.25 (0.26) when using the OD to 0.77 (0.55) when using ED. This corresponds to an improvement of approximately 208% and 111%, respectively. Besides the improved accuracy reached with the ED database, the results showed that the RF model provided better soil salinity estimations than the SVM model and that feature selection (i.e., Variance Inflation Factor-VIF and/or Genetic Algorithm-GA) increase both models´ reliability, with GA being the most efficient. This study highlights the potential of machine-learning and Sentinel-2 image combination for soil salinity monitoring in a data-scarce context, and shows the importance of both model and features selection for an optimum machine-learning set-up.

## 1. Introduction

### 1.1. Soil Salinity: A Global Issue

Soil salinization is one of the most important causes of soil degradation worldwide and represents a threat to the availability/sustainability of arable lands [1]. This process is naturally occurring in arid and semi-arid regions where low levels of precipitation and high evapotranspiration rates promote the concentration of salt in soils. In the ongoing climate change context, the global increase or decrease in temperature and precipitation are accelerating the soil salinization process in regions previously protected from this phenomenon. Besides climate, agricultural activity may substantially contribute to salt accumulation in soils. Indeed, irrigation without proper leaching and drainage leaves salt precipitates in the soil profile or on the ground surface [2]. Furthermore, over-irrigation can lead to a gradual rise of the groundwater level, which in turn favors soil surface salt accumulation due to capillary transport. Consequently, 33% of irrigated lands and 20% of croplands are affected by salinization [3,4,5] and 50% of global cropland areas will be affected by salinization by 2050 [6]. Finally, ongoing soil salinization spread is considered a major socioeconomic threat as it reduces agricultural productivity and quality [7], which in turn can lead to population outmigration [8].

### 1.2. Soil Salinity Monitoring: Remote Sensing and Machine-Learning Opportunities 

To deal with this problem, the first step is improving our ability to accurately monitor soil salinity changes in time and space. Traditionally, soil salinity data are obtained by conducting field surveys to collect soil samples for further laboratory soil electrical conductivity (EC) measurements. These methods are costly and time consuming, which considerably limit soil salinity data availability [9]. As soil salinity is highly affected by climate variations and/or agriculture practices, traditional monitoring methods are not suitable to follow soil salinization dynamics with a high spatial and temporal resolution. 

Since satellite optical images have a global coverage and a periodic revisit time, many authors investigated their ability to monitor soil salinity. Generally, soil salinity and/or vegetation indexes are derived from the combination of different spectral bands to train machine-learning models to retrieve soil salinity estimates [10,11,12,13,14,15,16,17,18,19]. With a higher spatial resolution than MODIS (500 m) and Landsat (30 m), and a revisit time of 5 days, Sentinel-2 (10 m) is particularly suitable for that purpose. For example, in Sri Lanka, a Partial Least Squares Regression (PLSR) model was set up to map soil salinity from Sentinel-2 images with satisfactory results (R^2^ = 0.69) [20]. In Iran, a Multi Linear Regression (MLR) model demonstrated a high accuracy (R^2^ = 0.75) for soil salinity estimates in the extremely saline region of Lake Urmia [21]. In China, a Cubist model was used to estimate soil salinity of an arid region with satisfactory results (R^2^ = 0.80) [17].

### 1.3. Which Machine-Learning Model Performs Best?

As different machine-learning models are available, some authors compared their reliability for soil salinity estimates to identify the most efficient one. For example, in Iran, Artificial Neural Network (ANN), Decision Tree (DT), Random Forest (RF) and Support Vector Machine (SVM) were compared to estimate salt content in soils during winter and summer seasons from Sentinel-2 images [22]. The authors showed that RF was the best model for predicting soil salinity during the summer season [22]. MLR, PLSR and RF models were compared to estimate soil salt content from Sentinel-2 images in Algeria. Overall, the RF model performed the best [23]. Convolutional Neural Network (CNN) and RF models were compared for soil salinity estimates across China and the results showed that the RF model was the most reliable [24]. In another study comparing the SVM, ANN and RF models to retrieve soil salinity estimates from Sentinel-2 images across Chinese arid areas, the SVM model reached the highest accuracy [10]. In northern Iran, Classification and Regression Trees (CART), RF, and SVM models were used on Sentinel-2 images and RF provided the most reliable estimates [25]. In southern Iran, after use of ANN, RF, SVM, PLSR and k-Nearest Neighbor (kNN) models [11], RF and SVM achieved the best soil salinity prediction accuracy. According to the above-mentioned studies, RF and SVM appear as the most efficient models to estimate soil salinity from Sentinel-2 images. 

### 1.4. Machine-Learning Training Set Weakness

Machine-learning model efficiency is sensitive to the database used to train the model. Typically, larger training sets result in better performance compared to smaller training sets [26,27]. The sensitivity of six machine-learning models (including SVM and RF) to different training set sizes was tested for Land Use Land Cover (LULC) classification in West Virginia, USA [27]. A total of nine training sets gathering 40, 80, 159, 315, 626, 1250, 2500, 5000 and 10,000 observations were used. The results revealed that SVM and RF global accuracy significantly increased with the number of samples, especially from the first to the fifth training sets, which had 40 and 626 samples, respectively. 

As soil salinity field measurements are costly and time consuming, most of the studies investigating machine-learning potential for soil salinity mapping relied on training sets gathering less than 100 samples [21,23,24,25,28,29]. In this context, it is crucial to increase the training set size in order to improve the reliability of machine-learning models to estimate soil salinity. 

### 1.5. Study Objectives

Based on the previously established state of the art, this study proposes a method to “artificially” increase the learning dataset size for machine-learning applied to soil salinity mapping in a data-scarce context. The RF and SVM models in combination with different feature selections were then used to test the method. This way, the study aims to provide a guideline towards the implementation of a consistent machine-learning set-up for soil salinity mapping in a data-scarce context. With extensive saline soil areas, the semiarid Bolivian Altiplano was chosen as the study area in order to improve regional soil salinity monitoring and thus support sustainable agriculture practices’ adaptation to face and mitigate the soil salinization process.

## 2. Materials 

### 2.1. Study Area 

**Figure 1:**
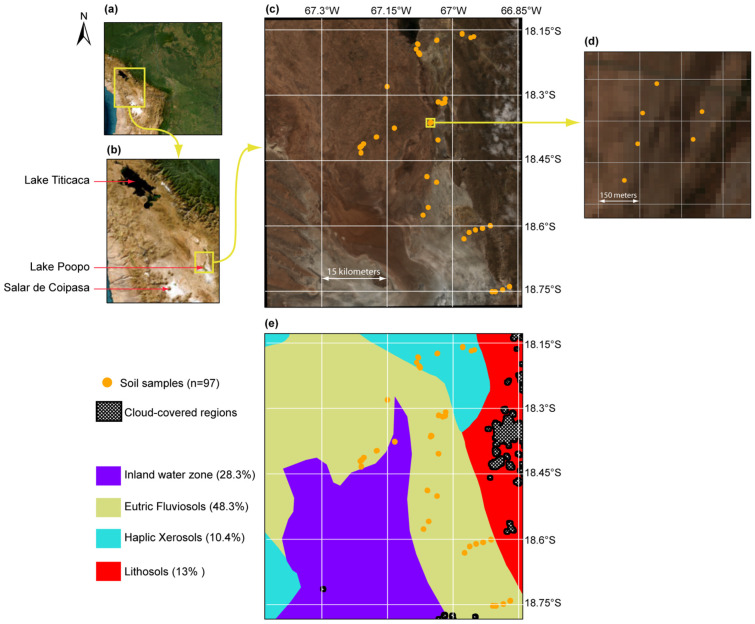
Location of the study area within the South American continent (**a**), within the Titicaca-Desaguadero-Poopó-Salar (TDPS) endorheic system (**b**), the location of the 97 soil samples around the lake Poopó (**c**,**d**) and the study area soil types according to the FAO soil map (**e**). Subplots (**c**,**d**) show the mean Sentinel RGB composition from the images obtained on 10 October, 15 October and 14 December in 2021 and subplot (**e**) indicates the area affected by cloud cover.

The study area is located in Bolivia between −66.84° to −67.43° longitude and −18.13° to −18.78° latitude, around Poopó Lake within the TDPS (standing for Titicaca, Desaguadero, Poopó and Salar of Coipasa) endorheic system. The TDPS system is located at a mean elevation of 4000 m.a.s.l [30]. In this system, water flows from north to south from Titicaca Lake to Poopó Lake through the Desaguadero River (Figure 1b). The only outflow of lake Poopó is the Lakajawira River in the southern end of the lake, which rarely flows towards the Coipasa Salar [31]. Thus, Poopó Lake could be seen as the terminal point of the TDPS water system. The climate of the Poopó Lake basin is arid with annual potential evapotranspiration and annual precipitation rates estimated at 1700 mm [32] and 400 mm [33], respectively. 

According to the FAO Digital Soil Map of the World (FAO-DSMW) the study region includes three different soil types (Lithosols, Eutric Fluvisols and Haplic Xerosols) along with an inland water zone (Figure 1e). Lithosols are characterized by their limited depth due to the constant presence of hard and coherent rocks, Fluvisols are developed from recent alluvial deposits, and Xerosols have an arid moisture regime. The endorheic and arid context promotes salt accumulation in soils, which is also facilitated by (1) the very flat topography preventing proper leaching and drainage of irrigation water and (2) the proximity of the groundwater level to the surface. As a result, the lake Poopó region holds extreme saline soils [2] and the salinization process is expected to worsen over time due to the ongoing desertification induced by climate change and human activity (mining, irrigation) [34,35]. 

### 2.2. Soil Sampling and Analysis

**Figure 2:**
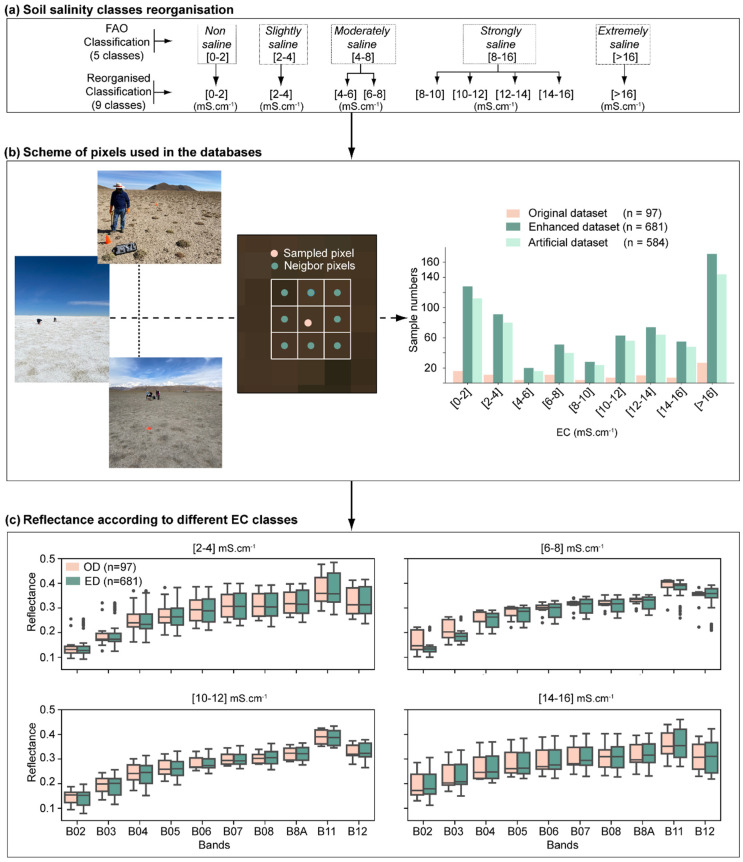
Method used for the development of the Original, Enhanced, and Artificial Databases: (**a**) reorganization of FAO salinity classes; (**b**) Scheme of pixels used in the databases and (**c**) its effect on the spectral response range for different EC classes.

Soil sample collection (n = 97) was carried out from 15–19 November 2021 (Figure 1c). Each sample was made up from 5 subsamples, each corresponding to a piece of soil of 20 × 20 cm^2^ with a depth of 20 cm, extracted at the corners and the center of a mesh of 10 × 10 m to take into account potential soil salinity heterogeneity [10]. The soil sampling locations were chosen based on local knowledge in order to cover the soil salinity range from low (<2 mS·cm^−1^) to high (>16 mS·cm^−1^) EC values over the study area (Figure 2b).

Soil electrical conductivity (EC) of the 97 samples was measured in the IIQ-UMSA laboratory (Instituto de Investigaciones Químicas, Universidad Mayor de San Andrés, La Paz, Bolivia) as a proxy for soil salinity. About 300 g of samples were dried at 40 °C in an oven, then disaggregated and sieved with a 2 mm mesh. Soil EC was measured using a 1:5 solid to liquid ratio method [36]. To do so, a sample of 5 ± 0.1 g of soil was placed in a polypropylene centrifuge tube and 25 ± 0.5 mL of deionized water (EC < 0.8 µS·cm^−1^) was added. Samples were agitated using a stirring table for 1 h and then left to decant for 30 min. EC (25 °C) was measured using a calibrated multi-parameter (HQ40D, Hach^®^, Loveland, CO, USA) equipped with a conductivity electrode (CDC401). The reproducibility of the method was assessed by measuring three contrasted soils in triplicate with low (2.3 ± 0.1 mS·cm^−1^), intermediate (16.7 ± 0.3 mS·cm^−1^) and high EC (29.9 ± 0.8 mS·cm^−1^). The analytical error (relative standard deviation) was lower than 3%. Additionally, at each site, triplicate soil samples spaced approximately 100 m apart were collected for EC analysis to evaluate spatial heterogeneity of soil salinity at the local scale (Figure 1c). The analysis of the triplicates showed a negligible EC difference at each site.

### 2.3. Sentinel-2 Images and Pre-Processing

Sentinel-2 images were obtained from the Copernicus website (https://apps.sentinel-hub.com/eo-browser) They are available at level 1C (Top Of Atmosphere—TOA) and level 2A (Bottom Of Atmosphere—BOA). The product 2A already includes an atmospheric correction process and was directly used in this study. The images captured by Sentinel-2 contain reflectance values in different bands at resolutions of 10, 20 and 60 m.

Sentinel-2 images and field samples were acquired at the end of the dry season in non-irrigated soils. In this season, meteorological conditions are homogeneous with scarce precipitation and no flood processes that could affect soil salinity concentration over time [37,38]. Therefore, soil salinity is not expected to change during the study period. Still, values from three Sentinel-2 images captured on 10 October, 15 October and 14 December of 2021, were averaged to minimize the effect of potential artefacts on the Sentinel-2 images that could be associated with local precipitation events and/or sensors and atmospheric correction uncertainties. These dates were chosen as they encompass the field campaign (15–19 November) and because the images´ metadata indicates cloud coverages of 7.8%, 9.7%, and 33%, respectively, with no cloud cover over the studied area. Actually, for each of the considered Sentinel-2 images, we used the “opaque clouds” and “cirrus clouds” mask bands to identify the pixels with cloud cover (i.e., opaque and/or cirrus). When a pixel was identified as cloud-covered in at least one of the considered Sentinel-2 images, this pixel was also considered as cloud-covered in the averaged image used for the analysis of soil salinity. Following this procedure, none of the pixels in which soil samples were extracted for the laboratory EC analysis, were identified as cloud-covered (Figure 1e). This guarantees no cloud cover influence on the signal observed by Sentinel-2, that therefore can totally be linked to soil surface and soil cover properties.

### 2.4. Machine-Learning Models

As reviewed in the introduction, SVM and RF models were used in the present study. Both models perform supervised learning for classification or regression controlled by different hyper-parameters [39]. The RF model uses a combination of tree predictors depending on a random vector sampled independently according to the same distribution [40], whereas the SVM model is based on the statistical learning theory [41,42]. 

For both models, all hyper-parameter values were set to default except the kernel for SVM that was set to polynomial degree three. This value was selected according to the principle of minimum error, on a trial-and-test analysis [10,13]. 

## 3. Methods

### 3.1. Elaboration of the Original Learning Database

For each of the 97 pixels including a soil sample, the reflectance values of the 10 bands of interest (B2, B3, B4, B5, B6, B7, B8, B8A, B11 and B12) were extracted from the averaged Sentinel-2 image. Since there are fewer bands of interest at 10 m spatial resolution (B2, B3, B4, B8), than at 20 m (B5, B6, B7, B8A, B11, B12), the bands at 10 m were resampled to 20 m using Snap tool and the nearest neighbor method [10], a technique for resampling raster data. Then, six (eleven) vegetation (salinity) indices and the Tasseled Cap Wetness (TCW) [10,11] were calculated from the selected bands (Table 1). 

This step led to the elaboration of the Original Database (OD) that includes soil EC values, reflectance of 10 bands and 16 indice values for the 97 Sentinel-2 pixels (n = 97).

### 3.2. Elaboration of the Enhanced Learning Database

As explained in the introduction, the efficiency of machine-learning models is sensitive to the training set size, with larger training sets resulting in better model performances [26,27]. In this context, a method to “artificially” expand the OD is proposed. The method is inspired by the sampling procedure used to set-up machine-learning databases for crop mapping. This method assigns the same type of crop for all the pixels included in the same agricultural field area [53,54,55,56,57]. This allows consideration of the spectral variability that can exist for the same type of crop due to the heterogeneous development of the crop inside the agricultural field. Following this path, we assumed that the eight pixels neighboring the pixel on which the EC measurement was performed are in the same EC class (Figure 2b). This hypothesis is supported by the similar topography, vegetation and soil characteristics around the sampling points (Figure 2b) and by the triplicate sampling made at each sampling point in the field showing low local (<100 m) soil EC variability (<%). 

In this context, the Enhanced Database (ED) was built by taking into account the 8 neighboring pixels of each of the 97 pixels of the OD (Figure 2b). In this process, the EC of the 8 neighboring pixels was equal to the one observed for the pixel in the middle (OD), whereas the 10 band reflectances and the 16 indice values were the ones observed at the 8 neighboring pixels’ respective locations. Therefore, this method allowed the consideration of the spectral variability (as denoted by the selected 10 bands) that exists for the same EC value (Figure 2c). Indeed, for the same EC class, the boxplot corresponding to each band was larger for ED than for OD (Figure 2c). 

The proposed method allowed the increase of the learning database size from n = 97 (OD) to n = 681 (ED) (Figure 2b). It should be noted that there should be 873 (97 × 9) observations in the ED database. Nevertheless, when two samples are very close to each other, one of them was discarded to avoid sharing neighboring pixels.

Being an adaptation of what is performed for LULC mapping, it is the first time that the proposed method has been implemented for soil salinity mapping. The method increased the size of the learning base by 700% without the need of additional field sampling, a very significant step forward in regions with scarce data where field surveys are limited due to a remote or sensitive socioeconomic context.

### 3.3. Elaboration of the Artificial Database

The Artificial Database (AD) only gathers the information corresponding to the neighboring pixels (n = 681 − 97 = 584). 

### 3.4. Comment on the Learning Databases

To guarantee consistency in the extrapolation of observed EC values to the neighboring pixels for the elaboration of ED (Section 3.2), EC classes are considered rather than numeric values. A total of nine EC classes (0–2; 2–4; 4–6; 6–8; 8–10; 10–12; 12–14; 14–16; >16 mS·cm^−1^) were defined for the three databases (i.e., OD, ED and AD). The classes were defined as a rearrangement of the Food and Agriculture Organization (FAO) classification [58], in order to have a bigger number of classes (Figure 2a). 

### 3.5. Machine-Learning Set-Up for Soil Salinity Estimation

One of the chosen models (RF or SVM) could be more reliable than the other for the region under study. Indeed, studies comparing RF and SVM reliability (along with other models) led to dissimilar conclusions according to the region. Using Sentinel-2 images as input, the SVM model was found to be more reliable than RF for soil salinity mapping in China [10], whereas the opposite was found in Iran [25]. In this context, the reliability of both models for the Poopó region was assessed. 

In addition, previous studies have highlighted that multicollinearity in the independent variables impacts the efficiency of machine-learning models due to input variable redundancy (i.e., not all the variables are necessary for the model) [10,11]. To minimize multicollinearity and optimize machine-learning models, two feature selection methods are mostly used: (1) the variance inflation factor (VIF) and (2) the genetic algorithm (GA) [10,12]. VIF assesses the correlation between variables to evidence redundancy and thus identifies some variables that could be eliminated. VIF is a pre-process to machine-learning models and therefore is insensitive to the model. GA attempts to optimize the combination of features by performing selections, crossovers and mutations on subsets of features, in order to improve the accuracy of the considered machine-learning models. Therefore, GA is sensitive to the model. 

In order to identify the most efficient scheme, four scenarios were investigated:

- scenario-1: using all variables
- scenario-2: applying VIF to all variables and selecting variables with VIF < 10
- scenario-3: applying the GA to all variables
- scenario-4: applying the GA to the variables obtained in scenario-2.

**Figure 3:**
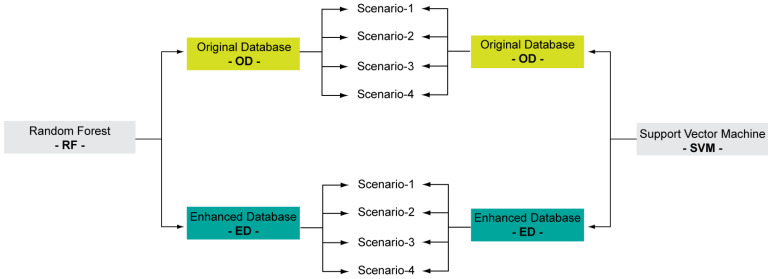
Flow-chart of the procedure to select the most efficient machine-learning set-up.

The four scenarios were tested for each model (RF and SVM) to evaluate their reliability using both OD and ED as learning datasets. Figure 3 summarizes the procedure to implement 16 simulations (2 models, 2 databases and 4 scenarios). 

As the learning databases rely on EC classes, SVM and RF classification algorithms were used. For every simulation, each database was randomly split into a training set (70%) and a validation set (30%). A seed was arbitrarily chosen to keep the same training and validation sets for all the simulations to ensure that the results would not depend on the internal randomness generator.

To assess the simulations’ accuracy, we calculated the confusion matrix and its associated recall, precision, F1 score, and overall accuracy (OA) as statistical indicators (Equations (1)–(4)). All statistical indicators are in the range 0 to 1, where 1 is the best value for classification.

(1) $$Recall=\frac{TP}{TP+FN}$$

(2) $$Precision=\frac{TP}{TP+FP}$$

(3) $$F1score=\frac{2\times Recall\times Precision}{Recall+Precision}$$

(4) $$OA=\frac{TP+TN}{TN+TP+FN+FP}$$

where TP, FP, FN and TN are the true positives, false positives, false negatives and true negatives, respectively.

### 3.6. Reliability Assessment of the Proposed Method

In order to assess how the data augmentation (i.e., to create ED) increases the models’ performance, the most efficient model (SVM or RF) was trained with AD (n = 584) and tested with OD (n = 97). In this process, the most efficient scenarios were used to consider the most efficient machine-learning set-up (see Section 3.4).

## 4. Results 

### 4.1. Feature Selection

The application of VIF to all variables (i.e., scenario-2) reduced multicollinearity by selecting four bands (B2, B4, B8, B11), three vegetation indices (NDVIre3, RDVI, WDVI), three soil salinity indices (S, S2, S6) and the TCW index as the most relevant variables (Table 2).

On the other hand, the application of GA to all variables (i.e., scenario-3) led to some differences in the variables selected for SVM and RF. The selected variables shared by both models included three bands (B2, B7, and B11), two vegetation indices (NDVIre2, NDVIre3) and three soil salinity indices (S, S1, S5). According to scenario-3, the SVM model was more sensitive to soil salinity indices than RF. Indeed, scenario-3 retained seven soil salinity indices for SVM and four for RF. 

When applying GA consecutively to VIF (i.e., scenario-4), the number of selected variables decreased substantially with only five and seven variables selected for SVM and RF models, respectively. Both models shared variables such as B11, S and S6 that appeared as the most relevant variables for soil salinity modelling based on machine-learning. 

**Figure 4:**
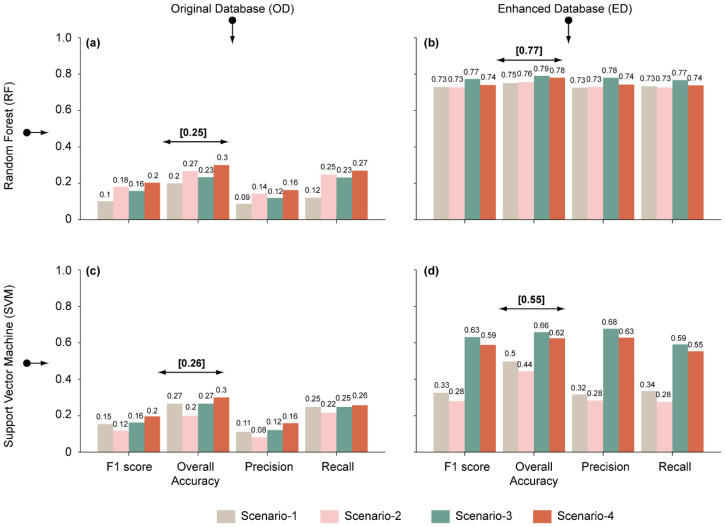
Models’ performances obtained in the validation step expressed in terms of F1-score, OA, precision and recall considering the validation dataset. (**a**,**c**) are the results based on the OD for RF and SVM models, respectively; (**b**,**d**) are the results based on ED for RF and SVM models, respectively.

Feature selection considerably decreased multicollinearity effects and improved the models’ efficiencies. For example, the metrics of the RF model (i.e., F1 score, OA, precision, recall) were higher for every scenario including a feature selection (scenario-2, -3 and -4) than for the scenario-1 which does not include it (Figure 4a,b). The same is true when considering the SVM model except for scenario-2, for which reliability decreases (Figure 4c,d). For the RF model, the benefits of the feature selection were bigger when the model was trained with a limited number of samples (i.e., OD, Figure 4a) than with a larger one (i.e., ED, Figure 4b), whereas the opposite is observed for the SVM model (Figure 4d,c). 

In summary, the application of feature selection resulted in a contrasting model reliability improvement, turning the feature selection into a model specific task. However, for both models, scenario-3 and -4 appear as the best feature selection option when using either a limited or a large sample training set (i.e., OD or ED), respectively (Figure 4). 

### 4.2. Benefits of the Proposed ED and Models Comparison

Figure 4 shows the accuracy of models obtained in the validation step in terms of F1 score, OA, precision and recall for each scenario. For both models and all scenarios, the use of ED led to higher model accuracy. For the RF model, the mean OA (as obtained for the four scenarios) jumped from 0.25 to 0.77 (308% increase) when using OD and ED as training datasets, respectively (Figure 4a,b). The improvement was smaller for the SVM model as mean OA increased from 0.26 to 0.55 using OD and ED, respectively (211% increase) (Figure 4c,d). 

**Figure 5:**
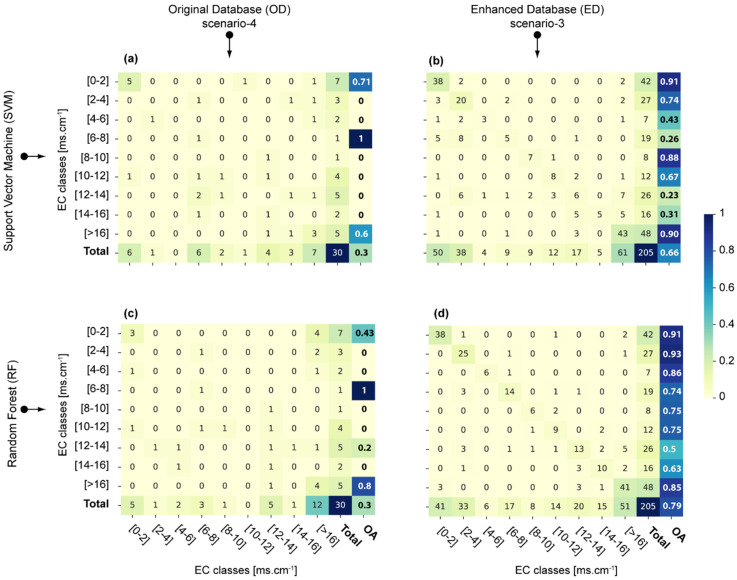
Confusion matrices with OA scores obtained in the validation step using the most efficient scenarios for both models and databases: (**a**) stands for the SVM applied to the OD using scenario-4, (**b**) SVM applied to the ED using scenario-3, (**c**) RF applied to the OD using scenario-4 and (**d**) RF applied to the ED using scenario-3.

Figure 5 shows the confusion matrices obtained in the validation step for both models (RF and SVM) and databases (OD and ED) considering the most efficient feature selection (scenarios-3 and -4). 

Due to the limited number of samples in the OD (n = 97), some EC classes have very few samples in the validation step. For example, (6–8) and (8–10) EC classes have only one sample (Figure 5a,c). In such cases, the accuracy of these classes can only be 1 or 0, thus preventing a consistent assessment of the model accuracy for those specific classes. When considering SVM and OD, the OA for the (6–8) and (8–10) classes is 1 and 0, respectively. 

When considering ED (n = 681) the (6–8) and (8–10) EC classes have 19 and 8 samples in the validation step (Figure 5b,d). This avoids uncertain binary results (i.e., 0 or 1). Consequently, contradictory results are observed when the same model is trained with OD or ED. For example, SVM appeared more (less) reliable for the (6–8) class than for the (8–10) class when trained with the OD (ED). Similarly, the classes with an OA of 0 for the RF model trained with OD presented OA values higher than 0.6 when using ED.

Finally, considering ED, RF presented higher OA values than SVM for all classes except the (8–10) and (>16) classes and therefore appeared as the best model for soil salinity mapping in the study region.

### 4.3. Reliability Assessment of the Proposed Method

**Figure 6:**
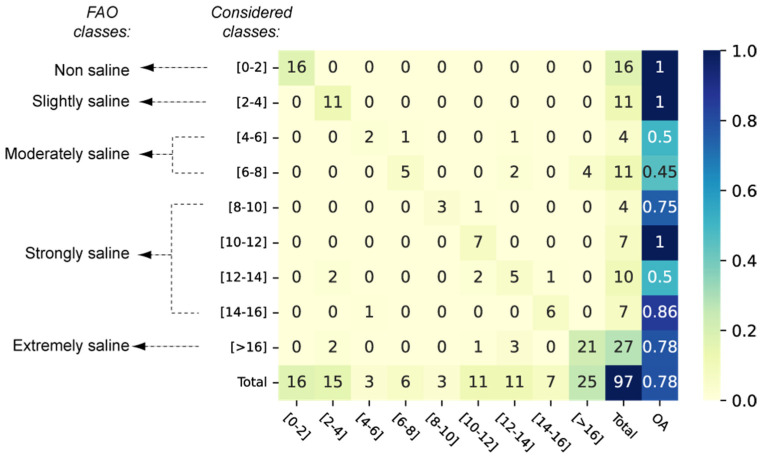
Confusion matrices with OA scores obtained in the validation step of the RF model trained with AD and validated with OD using scenario-3.

To assess the reliability of the proposed method, the AD (OD) is used as the training (validation) dataset for the most efficient model (i.e., RF). In this process, the most efficient feature selection (i.e., scenario-3) is used. Figure 6 shows the confusion matrix obtained in the validation step. The obtained OA of 0.78 confirmed that a machine-learning model trained with a database made of “artificial” observations (AD is only made of neighboring pixel information in which the EC was not measured) leads to consistent estimates of soil salinity. Therefore, the proposed method is an efficient alternative to overcome the difficulty of acquiring numerous field EC measurements. 

It is worth mentioning that even better metrics can be obtained by considering the FAO classes. For example, only three out of the seven errors of the classes ((8–10), (10–12), (12–14) and (14–16)) gathered into the strongly saline FAO class fall into the other FAO class (Figure 6). Therefore, the use of the wider-range FAO classification leads to an improved formal accuracy. 

### 4.4. Soil Salinity Map

**Figure 7:**
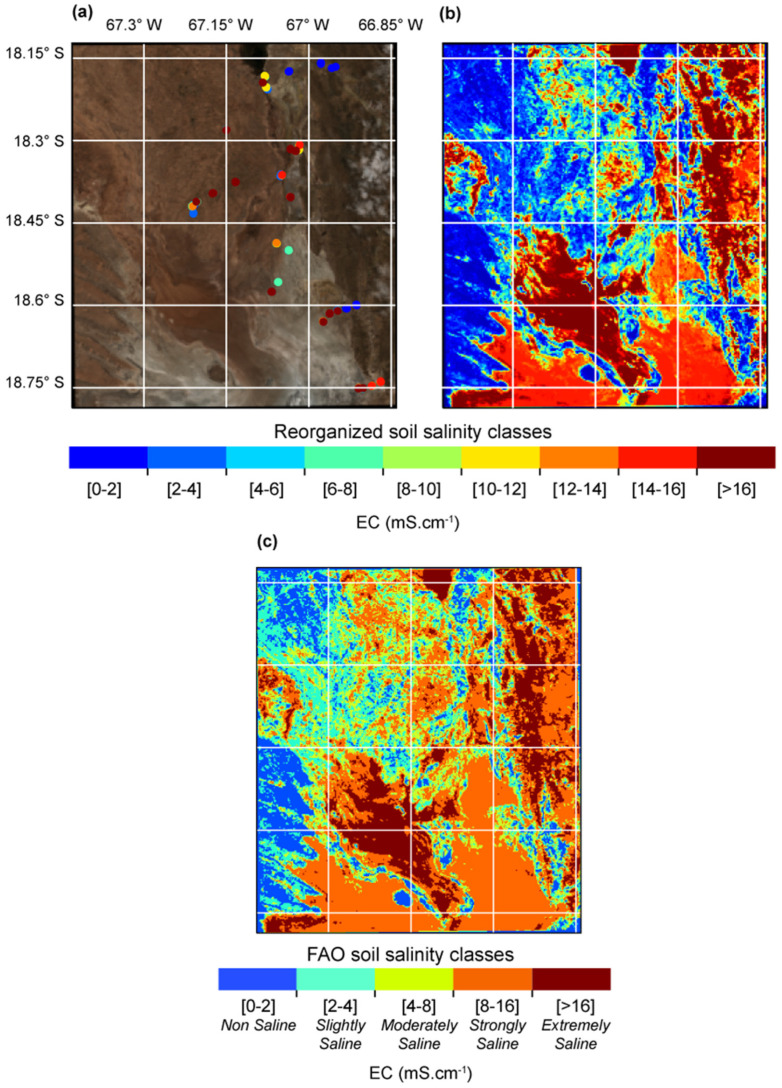
(**a**) Electrical conductivity (EC) obtained during the field campaign and (**b**,**c**) EC map obtained with the RF model trained with the ED on scenario-3 applied to the average Sentinel-2 images (10 October, 15 October and 14 December 2021).

Figure 7 shows the EC map obtained with the RF model with the ED dataset and scenario-3. The highest EC values (>16) are found in the southern and northern portions of the study area, in the lakes Poopó and Uru-Uru, respectively. This was expected as these lakes dried out during the field campaign and Sentinel-2 observations (November 2021), thus salt concentrated at these locations as a result of water evaporation. The southern part of the study area is more saline than the northern part as the southern Lake Poopó corresponds to the ending portion of the TDPS endorheic system (Figure 1a). Thus, saline water accumulation and subsequent evaporation are strong and tend to increase soil salinity content. Moreover, the southern part is located between lake Poopó and the Uyuni saltpan where the phreatic level is near the surface, therefore contributing to salt accumulation through an evaporative capillarity process.

The high EC values (>12) of the mountainous northeastern portion of the map is explained by the presence of cloud cover in the Sentinel-2 images used (Figure 1c) which may be spectrally confused with saline soils and by the non-consideration of topography features in the learning process. In fact, local topography could provide useful information to improve soil salinity mapping in the study region [37,38]. Actually, soils located in flat areas (i.e., the southern part) are more prone to salinization than soils situated in steep and rugged terrain, as soils in flat zones are closer to the aquifer level and more likely to accumulate water and thus concentrate salts due to evaporation [10,28,38]. Therefore, local topography could provide useful information to improve soil salinity mapping in the study region.

## 5. Discussion

The independent variables (i.e., regressors) considered in the study are limited to Sentinel-2 spectral bands and related vegetation/salinity indexes. Easily accessible, they are commonly used in similar studies [10,11,12,13,14,15,16,17,18,19]. 

However, topography features also affect the soil salinity level [37,38] and could have been used to improve the models´ accuracy. For example, a study in Mongolia shows that topography (and groundwater depth) controlled the soil salinity distribution [38]. Actually, soil salinity is high (low) in areas with lower elevations (with more undulating terrain) and tends to decrease with the increase in elevation [9,59]. In China, high soil salinity contents were found in flat landscapes and shallow groundwater depths, whereas low soil salinity contents were found in areas with large topographic changes [10,28]. The topographic effect reinforced by local climate conditions. Indeed, regions at low elevation are warmer than regions at high elevation which favors stronger evaporation rates that in turn favor soluble salts to accumulate on the soil surface [60]. Flat regions are also prone to strong local winds that increase evaporation and favor salt accumulation at the soil surface [28]. In that context, a study in the Chinese Ebinur Lake basin showed that along with Sentinel-2 bands and soil salinity and vegetation indices, elevation is an important variable for soil salinity mapping [61]. Another study in the Chinese Ogan-Kuqa River Oasis showed that topographic indices are the second most important predictor variables for soil salinity mapping [62]. As shown in Figure 7, high soil salinity values are found in the eastern part of the study region that include high elevated and undulating terrain. This unexpected feature could be corrected in future studies by considering topography indices along with climate variable models as regressors. 

In addition to topography and climate, groundwater level and agricultural activities also affect the soil salinity level [37,38]. However, this information is unavailable or scarce in remote regions. In that sense, Sentinel-1 radar polarization could provide useful information to improve model reliability. Indeed, Sentinel-1 shows a great potential for soil humidity mapping [63,64,65,66] and to follow irrigation practices [67,68,69]. Therefore, Sentinel-1 gathered valuable information for soil discretization according to its exposure to irrigation practices and groundwater capillarity, both favoring salt accumulation through the evaporation process. Following this path, recent studies highlighted the potential of Sentinel-1 data for soil salinity mapping [13,70,71]. Some authors already assessed the potential of merging Sentinel-2 and Sentinel-1 for soil salinity mapping in different regions [62,72]. In fact, the combination of Sentinel-2 and Sentinel-1 data improved soil salinity model estimates in comparison to the Sentinel-2 alone [62]. These studies could be used as a guideline to merge Sentinel-2 and Sentinel-1 data to improve soil salinity mapping in the TDPS. 

Finally, to guarantee consistency in the extrapolation of the observed EC to neighboring pixels, the proposed method is limited to regions with homogeneous LULC, soils and topography features at the pixel-to-pixel scale. To strengthen consistency in the EC extrapolation step, EC classes had to be considered (i.e., discreet values) instead of continuous values, to take into account small EC variations that might occur at the pixel-to-pixel scale. Therefore, the use of the ED is limited to classification models. Still, the consideration of nine EC classes (0–2, 2–4, 4–6, 6–8, 8–10, 10–12, 12–14 14–16 and >16) provide a detailed description of soil salinity spatial distribution (Figure 7). In a data-scarce context, such a level of information is valuable for soil management and agriculture adaptation to soil salinity hazard. 

## 6. Summary and Conclusions

This study proposes a simple method to expand the learning database without the need for additional field campaign observations. The performance of the method as compared to a “traditional” approach was assessed with two machine-learning models (i.e., Random Forest-RF and Support Vector Machine-SVM) implemented in the lake Poopó region, located in the South American Altiplano TDPS system. The main results of the study can be summarized as follows:

- The proposed method allowed to expand from a learning database of 97 field observations to 681 observations (a 700% increase);
- The use of the enhanced database (ED) significantly improved the model accuracy to estimate soil salinity, resulting in significantly better metrics for both models (i.e., RF and SVM) than when using the original database (OD);
- The improvements in performance obtained with the proposed method were better when considering the Electrical Conductivity (EC) classes. Indeed, for some EC classes, the number of observations in the OD were too low to correctly train and validate the models. The use of the ED allowed the overcoming of this problem by significantly increasing the number of observations in all classes;
- In the case of the limited training dataset (i.e., OD), the Genetic Algorithm (GA) led to more reliable model prediction than the use of Variance Inflation Factor (VIF) for the SVM model whereas the opposite was true for the RF model.
- In the case of the larger training dataset (i.e., ED). The GA led to more reliable model predictions than the VIF for both models.
- Overall, the RF model is more suitable than SVM for soil salinity mapping in the arid Lake Poopó region.

The method described in this paper provided a cost and time-efficient approach to increase the learning database, which in turn resulted in a greater improvement in the accuracy of the Random Forest and Support Vector Machine models. This is particularly relevant for soil salinity mapping in regions where the combined effect of a remote location and a difficult socioeconomic context prevents frequent field sampling at high spatial resolution for soil salinity measurements. Future applications using Sentinel-1 radar images could lead to even better performances of the machine-learning and thus contribute to reliable up-to-date monitoring of soil salinization in remote arid regions.

## Figures and Tables

**Table 1:** Sentinel-2 bands and related indices used for model training and validation.

Acronym	Definition	Reference
B2-Blue, B3-Green, B4-Red, B5-Rededge1, B6-Rededge2, B7-Rededge3, B8-NIR, B8A-Rededge4, B11-SWIR1, B12-SWIR2	Sentinel-2 bands	
Salinity Index 1 (SI)	$\sqrt{B2\times B4}$	[14,43]
Salinity Index 2 (SI1)	$\sqrt{B3\times B4}$	[14,43]
Salinity Index 3 (SI2)	$\sqrt{B3^{2}+B4^{2}+B8A^{2}}$	[14,43]
Salinity Index 4 (SI3)	$\sqrt{B3^{2}+B4^{2}}$	[14,43]
Salinity Index 5 (S)	B4/B8A	[44]
Salinity Index (S1)	B2/B4	[45]
Salinity Index (S2)	$\left(B2-B4\right)/\left(B2+B4\right)$	[45]
Salinity Index (S3)	B3×B4/B2	[45]
Salinity Index (S5)	B2×B4/B3	[45]
Salinity Index (S6)	B4×B8A/B3	[45]
Normalized Difference Salinity Index (NDSI)	$\left(B4-B8A\right)/\left(B4+B8A\right)$	[45]
Normalized Difference Vegetation Index (NDVI)	$\left(B8A-B4\right)/\left(B4+B8A\right)$	[46]
Normalized Difference Vegetation Index red-edge 1 (NDVIre1)	$\left(B8A-B5\right)/\left(B5+B8A\right)$	[47]
Normalized Difference Vegetation Index red-edge 2 (NDVIre2)	$\left(B8A-B6\right)/\left(B6+B8A\right)$	[47]
Normalized Difference Vegetation Index red-edge 2 (NDVIre3)	$\left(B8A-B7\right)/\left(B7+B8A\right)$	[48]
Renormalized Difference Vegetation Index (RDVI)	$\left(B8A-B4\right)/\left(\sqrt{B8A+B4}\right)$	[49]
Weighted difference vegetation index (WDVI)	B8A−0.5×B4	[50,51]
Tasseled cap wetness (TCW)	0.1509×B2+0.1973×B3+0.3272×B4+0.3406×B8−0.7112×B11−0.4573×B12	[52]

**Table 2:** Selected variables per scenario and model.

Model	Scenario	Variables
SVM, RF	scenario-1	B2, B3, B4, B5, B6, B7, B8, B11, B12, B8A, NDSI, NDVI, NDVIre1, NDVIre2, NDVIre3, RDVI, S, S1, S2, S3, S5, S6, SI, SI1, SI2, SI3, TCW, WDVI.
	scenario-2	B2, B4, B8, B11, NDVIre3, RDVI, S, S2, S6, TCW, WDVI.
SVM	scenario-3	B2, B4, B5, B7, B11, NDVI, NDVIre2, NDVIre3, RDVI, S, S1, S3, S5, S6, SI1, SI2.
	scenario-4	B2, B4, B11, S, S6.
RF	scenario-3	B2, B6, B7, B8, B11, B12, NDSI, NDVIre2, NDVIre3, S, S1, S2, S5, TCW, WDVI.
	scenario-4	B8, B11, NDVIre3, S, S2, S6, TCW.

## Data Availability

Data are contained within the article.
